# Molecular dynamics simulation on regulation of liquid–liquid phase separation of repetitive peptides

**DOI:** 10.1038/s41598-024-64327-7

**Published:** 2024-06-11

**Authors:** Xiaojun Yang, Yanwei Wang, Guangcan Yang

**Affiliations:** Department of Physics, Wenzhou University, Wenzhou, 325035 China

**Keywords:** GENESIS, Molecular dynamics, Liquid–liquid phase separation, Polypeptides, Electrostatic interaction, Hydrophobicity, Computational biophysics, Intrinsically disordered proteins, Molecular biophysics

## Abstract

Understanding the intricate interactions governing protein and peptide behavior in liquid–liquid phase separation (LLPS) is crucial for unraveling biological functions and dysfunctions. This study employs a residue-leveled coarse-grained molecular dynamics approach to simulate the phase separation of repetitive polyproline and polyarginine peptides (poly PR) with varying lengths and sequences in solution, considering different concentrations and temperatures. Our findings highlight the crucial role of sequence order in promoting LLPS in peptides with identical lengths of repetitive sequences. Interestingly, repetitive peptides containing fewer than 10 polyarginine repeats exhibit no LLPS, even at salt concentrations up to 3 M. Notably, our simulations align with experimental observations, pinpointing a salt concentration of 2.7 M for PR25-induced LLPS. Utilizing the same methodology, we predict the required salt concentrations for LLPS induction as 1.2 M, 1.5 M, and 2.7 M for PR12, PR15, and PR35, respectively. These predictions demonstrate good agreement with experimental results. Extending our investigation to include the peptide glutamine and arginine (GR15) in DNA solution, our simulations mirror experimental observations of phase separation. To unveil the molecular forces steering peptide phase separation, we introduce a dielectric constant modifier and hydrophobicity disruptor into poly PR systems. Our coarse-grained analysis includes an examination of temperature effects, leading to the inference that both hydrophobic and electrostatic interactions drive phase separation in peptide systems.

## Introduction

Liquid–liquid phase separation (LLPS) is a prevalent and reversible phenomenon in biological systems, driven by the coordinated condensation of proteins and nucleic acids. This intricate process leads to the formation of membraneless cellular organelles (MLO) and plays crucial roles in physiological functions such as DNA repair^1,2^ and gene expression regulation^3^. Additionally, LLPS is implicated in the pathogenesis of diseases, including amyotrophic lateral sclerosis (ALS) and frontotemporal dementia (FTD)^2,4^. Recently study shows that some specific region containing sequence tendency for the Interaction between Low-Complexity Intrinsically Disordered Proteins is essential for LLPS^5^.

The thermodynamic process of LLPS involves proteins minimizing their free energy through weak interactions, resulting in the separation into dilute and concentrated phases. Balancing entropy and enthalpy forces is a complex challenge, driven by electrostatic and hydrophobic interactions, encompassing charge–charge, cation–π, dipole–dipole, and π–π molecular interactions^6^.

Recently, several coarse-grained simulations have shed light on various aspects of Liquid–Liquid Phase Separation (LLPS). These include the dynamics surrounding lipopolysaccharide (LPS)-containing outer membranes^7^, as well as the modeling of disordered biomolecules for LLPS simulations^8^. The advantages of employing simple coarse-grained models have been further examined and contrasted with LLPS propensities^9^. Moreover, there has been significant progress in developing a transferable coarse-grained model to evaluate the impact of Post-Translational Modifications (PTMs) on LLPS within a computational simulation framework^10^.

Repetitive peptides involved in LLPS are not only implicated in LLPS itself but also play crucial roles in various biological functions. A significant illustration of this is found in the identification of C9ORF72 as a causal gene for familial amyotrophic lateral sclerosis and frontotemporal dementia^11,12^. The mutations of C9ORF72 are prevalent in both genetic forms of these debilitating diseases^12^. Patients with C9ORF72 mutations exhibit expanded six-nucleotide repeats, activating unconventional translation mechanisms and producing dipeptide repeat proteins, including poly PR and poly GR, known for their notable toxicity in cellular and animal models^13–15^.

In our present study, we employ MD simulations to investigate the effects of LLPS on peptide polymerization length, salt concentration, and temperature. Experimental settings include the introduction of dielectric constant modifiers and hydrophobic disruptors to modify the interaction between poly-PR^16^. In simulations, we manipulate the amount of charge and hydrophobicity of amino acids. Our findings reveal the crucial role of higher salt concentrations in LLPS when the repeat proline and arginine (PR) peptide exceeds a certain threshold. The exploration of repeat PR sequence regulation holds promise for gaining insights into ALS mechanisms and inspiring potential therapeutic strategies.

## Materials and methods

### Simulation model

In our simulation, we use Hydropathy Scale (HPS) Coarse-Grained (CG) model for the LLPS of the repetitive peptides^17^. The Hydropathy Scale (HPS) model specifies residue-specific features such as mass and spring constants, also including electrostatic and hydrophobic interactions. This model uses a bead representation for each residue, allowing for sequence dependence and is also suitable for simulations on large time and length scales. It links the strength of short-range interactions to an existing hydrophobicity scale and ties the strength of electrostatic interactions to the total charge carried by the shielding residues. It is a coarse-grained polymer model with one site per residue, linking interactions between residues to a hydropathy scale ranging from 0 to 1. In our simulations, we use the velocity Verlet integrator specifically for coarse-grained simulations, with a total simulation step of 1,000,000, a timestep of 0.01 ps, and both energy output intervals and trajectory output intervals set at 10,000.

### Force field and truncated region

In molecular dynamics simulations, the GENESIS platform utilizes a truncation distance to delineate the force range (force field) of the current atoms. Specifically, the truncation distance defines the interaction between atoms with a distance less than the specified value during the calculation of atom interactions. This is crucial because the interaction between atoms diminishes rapidly with increasing distance, and interactions beyond a certain range can be considered infinitesimally small and, therefore, negligible.

Within the GENESIS platform, users typically predefine the truncation distance, selecting it based on the size and nature of the system. During simulation, the GENESIS platform determines the interaction range around the current atom based on the specified truncation distance, calculating interactions only between atoms within a distance less than the truncation distance. This approach significantly reduces computational load and enhances simulation efficiency.

It’s important to emphasize that the selection of an appropriate truncation distance profoundly influences the accuracy and reliability of simulation results. Opting for a truncation distance that is too small may lead to an insufficient interaction range, introducing potential bias in simulation outcomes. On the other hand, choosing a truncation distance that is too large can result in an overly broad interaction range, escalating computational complexity and simulation time. Hence, selecting an appropriate truncation distance is a critical consideration in simulation. Table 1 outlines the key parameters associated with the truncation distance.

**Table 1:** Default truncation parameters.

Nonbonded term	Cutoff (${\text{r}}_{\text{C}},\dot{\text{A}}$)	Neighbor list distance (${\text{r}}_{\text{p}},\dot{\text{A}})$
${\text{E}}_{\text{ele}}$	52	57
${\text{E}}_{\text{HPS, KH}}$	39	44
${\text{E}}_{\text{bp}}$	18	23
${\text{E}}_{\text{PWMcos}}$ and ${\text{E}}_{\text{HB}}$	–	23
${\text{E}}_{\text{exv}}^{(1)}$ and ${\text{E}}_{\text{exv}}^{(2)}$	–	15

### Data analysis

We employed the recently developed CG model HPS to simulate the dynamics of the PR series peptides using GENESIS. The simulations conducted with GENESIS resulted in the generation of DCD (Dynamical Coordinates Dump) and PDB (Protein Data Bank) files, both of which are standard file formats containing molecular structure information commonly obtained from simulations or experiments in molecular biology and chemistry.

Consequently, by utilizing DCD and PDB files, we can easily analyze atomic concentration. The analysis was carried out using the Origin software, facilitating a detailed examination of atomic concentration patterns.

## Results and discussion

### Simulation of poly PR with different polymerization lengths in salt solution

**Figure 1:**
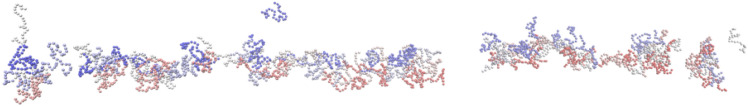
120 PR15 chains in the initial state.

GENESIS allows atoms and molecules to interact for a fixed period, giving a dynamic “evolution” view of the system. We set multiple ion strength gradients to observe the evolution of the system. We initialized 120 poly-PRs and placed them in a box with a periodic boundary condition of 180 × 180 × 1800 A^3^. In the conducted simulation, we utilized a slab-geometry model with a substantial aspect ratio. The system was confined within a three-dimensional box that incorporated periodic boundary conditions along two axes, while the third axis included a free, or alternatively a reflective, boundary. The slab sampling method is particularly suitable for Liquid–Liquid Phase Separation (LLPS) simulation because it allows for the study of interfacial phenomena, which are often prevalent in phase separation processes^17^. Figure 1 shows the initial state of 120 PR15 chains in the box.

To enhance computational efficiency, GENESIS utilizes the ATDYN module, which employs the MPI (Message Passing Interface) and OpenMP protocols (mixed MPI + OpenMP) to harness the capabilities of multiple CPU cores. MPI is generally employed for communication between different machines, nodes, or processors, where memory is not shared across these entities (distributed memory). Conversely, OpenMP is utilized within a single processor, making use of shared memory in parallel computing.

In our simulations, we configured 16 MPI processes and 4 OpenMP threads, resulting in a total of 64 CPU cores. The simulation involved studying the LLPS phenomenon of poly PR with varying polymerization lengths (PR2, PR4, PR8, PR9, PR10, PR11, PR12, PR15, PR25, and PR35) in response to changes in KCl concentration from 0 to 3000 mM.

All simulation parameters were kept consistent across the experiments. The initial hydrophobic values of proline and arginine were referenced from the literature^17^. As the salt concentration increased, the hydrophobicity of arginine also increased, leading to an elevation in its hydrophobic value^6^.

**Figure 2:**
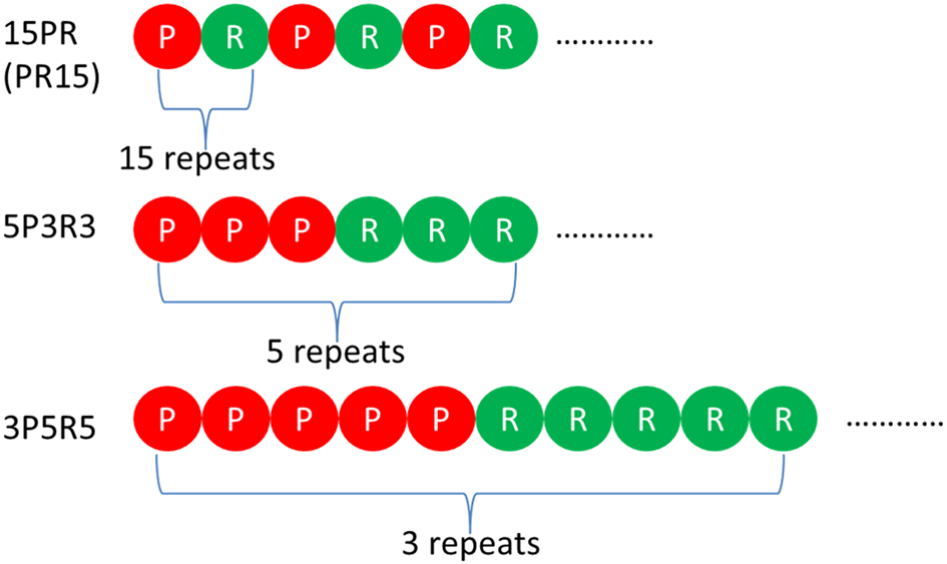
Schematics of PR ordering.

**Figure 3:**
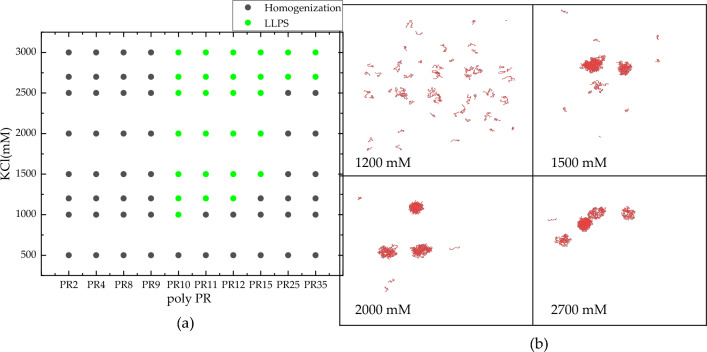
(**a**) The dependence of liquid–liquid separation range for PR series repetitive peptides at various KCl concentrations. Grey dots indicate no phase separation, while green dots indicate LLPS. (**b**) PR15 results at concentrations of 1200 mM, 1500 mM, 2000 mM, and 2700 mM potassium chloride in sequence.

The arrangement of amino acid residues in peptides is illustrated as a cartoon in Fig. 2. Figure 3 shows the dependence of liquid–liquid separation of PR series on their length and KCl concentrations, as well as the simulation results of PR15 at KCl concentrations of 1200 mM, 1500 mM, 2000 mM, and 2700 mM. The simulation results are shown in Fig. 3a that no phase separation occurred when the number of repeat sequences in poly PR was small. In the region where phase separation could occur, an increase in the number of repeat sequences in poly PR correlated with an increased salt concentration required for LLPS.

As shown in Fig. 3b, PR15, a representative peptide in our study, displayed dispersion at concentrations below 1500 mM. Upon reaching 1500 mM salt concentration, simulation results revealed aggregation and further increments in salt concentration maintained PR15 in an aggregated state. Notably, PR2, PR4, PR8, and PR9 did not exhibit significant aggregation at either low or high salt concentrations. Aggregation was observed only when the polymer length increased to PR10. Poly PR peptides demonstrated even distribution in low salt conditions, with aggregation effects becoming prominent in high salt concentrations. Experimental findings corroborated varying degrees of aggregation under different potassium chloride ion concentrations.

In low-concentration conditions, the aggregation of multichain proteins tended to be weaker than in high-concentration conditions. This phenomenon can be attributed to the interplay of hydrophobic and electrostatic interactions among the peptides. In low salt conditions, electrostatic repulsion between positively charged arginine residues hindered LLPS, despite the presence of hydrophobic attraction between peptides. Conversely, high salt concentrations reduced electrostatic repulsion between Arg-Arg pairs in low salt conditions, resulting in weak mutual attraction^6^.

As the polymer length increased, PR25 and PR35 exhibited LLPS behavior in critical-concentration KCl solutions while maintaining a homogeneous state below the critical concentration. In comparison, shorter chains like PR4 and PR8, with fewer interaction sites, were unable to achieve phase separation behavior^16^.

**Figure 4:**
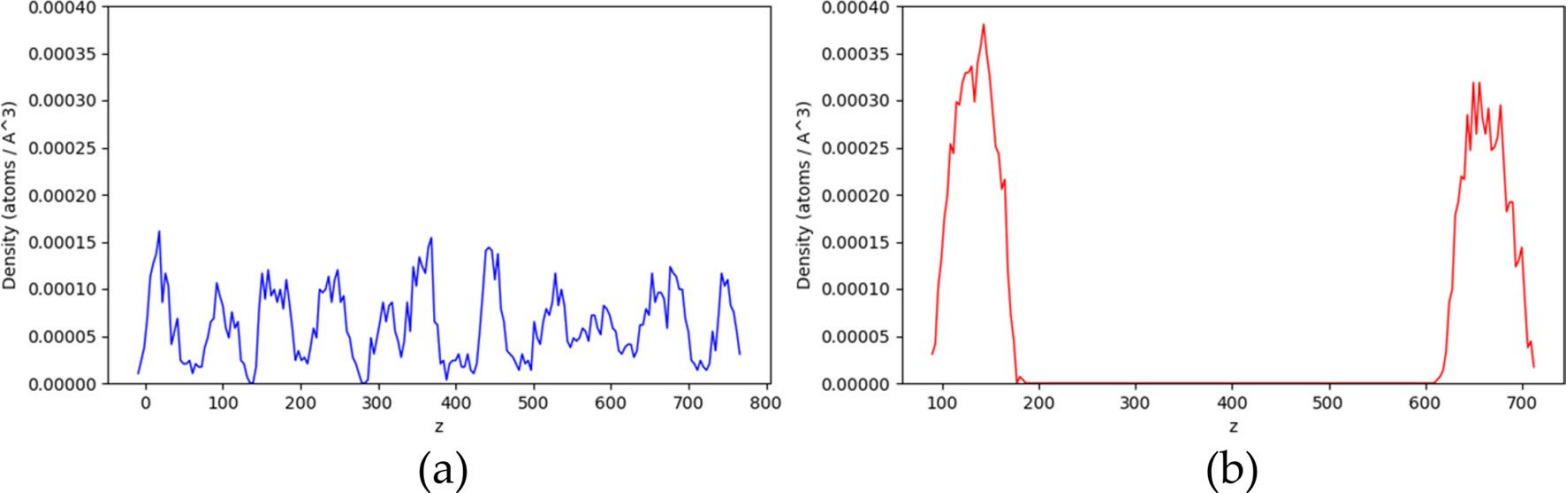
The density profile of PR15 at 2700 mM KCl in z direction. (**a**) The initial state of simulation. (**b**) The final state of simulation.

**Figure 5:**
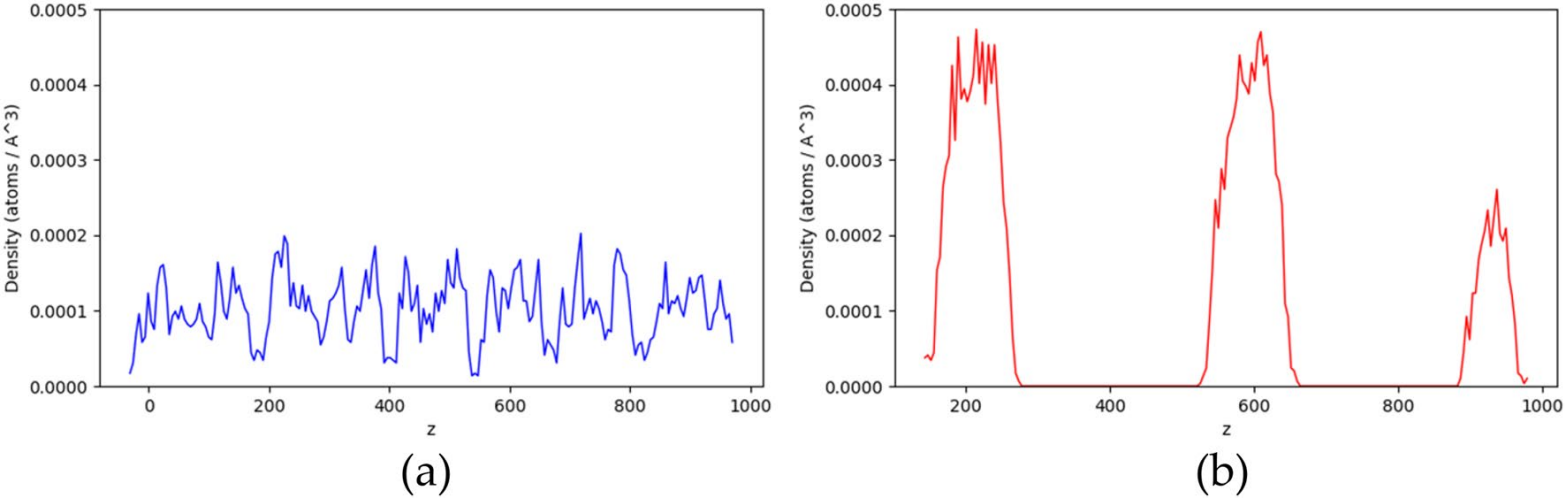
The density profile of PR25 at 2700 mM KCl in z direction. (**a**) The initial state of simulation. (**b**) The final state of simulation.

In addition of the simulation snapshots, we added density profile analysis in the revised version. The phase separation process can often be characterized via the analysis of density profiles. The density profiles are presented in Figs. 4 and 5. We can see that a sharp change in the density profile usually indicates the phase boundary, corresponding to LLPS.

**Figure 6:**
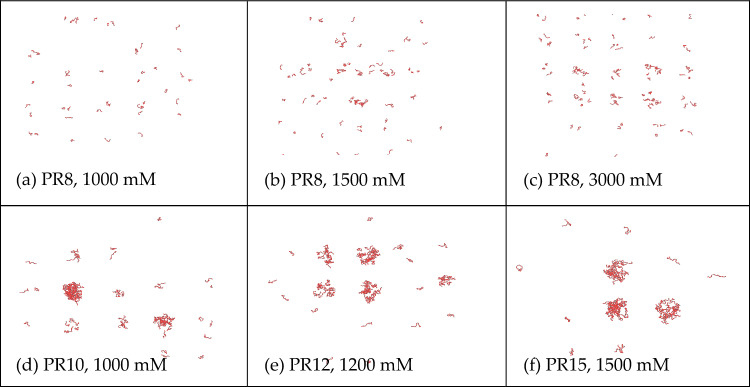
The simulation results under the condition of maintaining a total arginine count of 1200, that is, 150 chains for PR8, 120 chains for PR10, 100 chains for PR12, and 80 chains for PR15. Among them, PR10, PR12, and PR15 are the simulations that just occur at the salt concentration where phase separation happens.

To decouple the effect of salt screening and (PR) group repetitivenes, we accomplished the molecular dynamics simulations with a fixing total number of arginine, as illustrated in Fig. 6. The simulations were performed for PR8, PR10, PR12, and PR15, under conditions where the total arginine count was fixed at 1200, resulting in 150 chains for PR8, 120 for PR10, 100 for PR12, and 80 for PR15. From the simulation outcome, it is notable that PR8 did not undergo phase separation in any of the salt concentrations, while PR10, PR12, and PR15 experienced phase separation at salt concentrations of 1000 mM, 1200 mM, and 1500 mM respectively. This coincides with the results of the simulations that kept the chain numbers constant. Therefore, it can be concluded that the occurrence of PR phase separation is associated with both the salt concentration and its length, while variations in PR count do not impact phase separation results.

### Simulation of the regulation of poly PR by hydrophobic and electrostatic interactions

**Figure 7:**
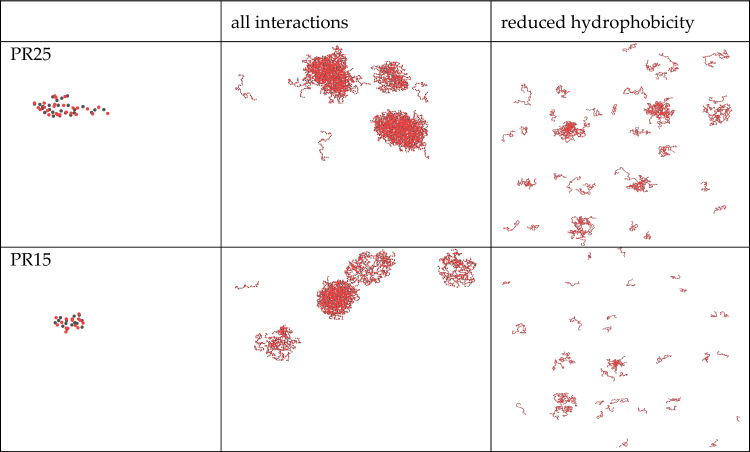
LLPS simulation of PR15 and PR25 at 2700 mM salt concentration, where blue represents proline and red represents arginine.

For PR15, phase separation occurs at 1500 mM, and in our experiments, we modulate its hydrophobic and electrostatic interactions by introducing dielectric constant disruptors such as 1, 6 hexanediol and anhydrous ethanol^3,16,18^. The program’s parameter file employs the default parameter set suggested by Tesei et al.^19^ in the latest version of GENESIS-CG-tool, but it can also be customized to other parameter sets^17,20,21^. However, such changes do not impact our analysis of the experimental results, as the underlying principles remain consistent. In our simulations, we manipulate a single variable by adjusting the hydrophobic value of arginine while keeping the hydrophobic value of the proline constant (set to λ = 1). This adjustment simulates the addition of dielectric constant disruptors to regulate the hydrophobic effect, mirroring the experimental conditions. We continue our investigation using PR15 and PR25 as the focal peptides, adjusting the hydrophobic value of arginine in a 2700 mM salt solution until the aggregates disperse, effectively disrupting their hydrophobic interactions. The simulation results are shown in Fig. 7.

**Figure 8:**
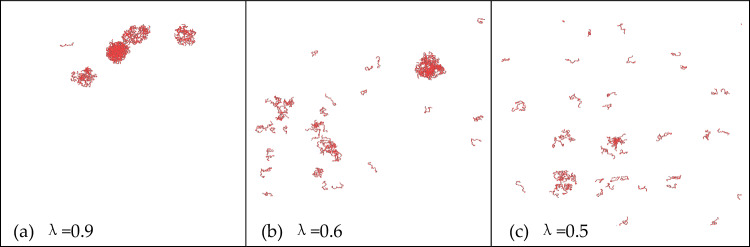
LLPS behavior of PR15 under different hydrophobic values of arginine.

Continuing our analysis, we delve deeper into the phase separation behavior of PR15. Figure 8 displays the LLPS behavior of PR15 at different hydrophobic values of arginine. In Fig. 8a, PR15 is depicted at 2700 mM, where its proline and arginine hydro-phobic values are set to λ = 1.0 and λ = 0.9, respectively. Moving to Fig. 8b, we observe the impact of altering the arginine hydrophobic value to 0.6 while maintaining the proline hydrophobic value constant. Figure 8c illustrates that a further reduction in the arginine hydrophobic value to 0.5 results in the complete dissolution of the PR15 aggregate, effectively destroying the phase separation. This outcome underscores the pivotal role of hydrophobic interactions in the LLPS of PR peptides. In our simulation, we disrupted phase separation by diminishing the hydrophobic value of arginine, mirroring real-world experiments where phase-separated solutions were disrupted by introducing hydrophobic disruptors^16^. The fundamental principle underlying both methods remains consistent.

**Figure 9:**
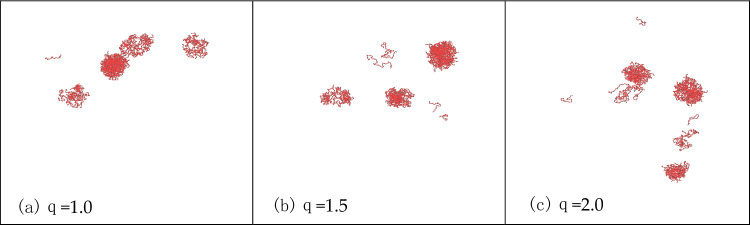
LLPS behavior of PR15 (2700 mM salt concentration) under different electrostatic forces.

Moving forward, we examine the influence of electrostatic interactions on phase separation. Given that proline is electrically neutral and arginine carries a positive charge, electrostatic interactions predominantly occur between arginine and arginine. These interactions are governed by the Debye–Hückel theory, with the Debye–Hückel formula elaborated in reference^17^. The crucial aspect is understanding how the electrostatic force changes when the charge is modified. Assuming E is the electrostatic force calculated with one charge of arginine, setting the charge to 2 results in the electrostatic force becoming 4 times the original, representing electrostatic repulsion. In our simulation, we set the charge of arginine to 1.5 and 2, corresponding to electrostatic repulsions 2.25 times and 4 times the original, respectively. Figure 8 shows the LLPS behavior of PR15 at a salt concentration of 2700 mM under different electrostatic forces. As depicted in the Fig. 9, the electrostatic force exhibits no significant impact on the aggregation state of PR15, indicating that electrostatic interactions have a minor effect on LLPS. In the absence of alterations in hydrophobic interactions, the influence of electrostatic interactions on phase separation is minimal, aligning with existing experimental conclusions^16^.

### The effect of temperature on phase separation

**Figure 10:**
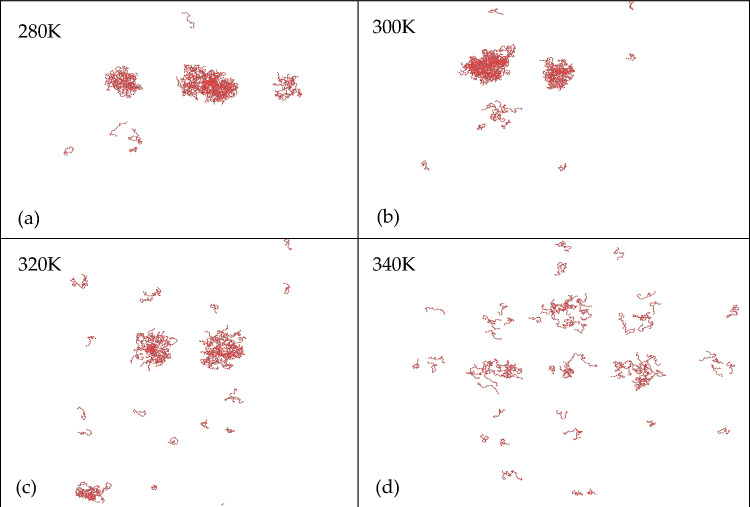
LLPS behavior of PR15 (1500 mM salt concentration) at different temperatures.

Furthermore, we investigated the impact of temperature on LLPS simulating the phase separation of PR15 at various temperatures under conditions of 1500 mM. Figure 10 shows the impact of different temperatures on the LLPS behavior of PR15 at a salt concentration of 1500 mM. The results indicate that an increase in temperature impedes the LLPS of PR15. This effect can be attributed to the temperature-induced alteration in the conformational entropy of the polypeptide, which hinders the aggregation of polypeptides and inhibits the occurrence of LLPS^22,23^.

### The effect of different arranging orders of poly PR on phase separation

**Figure 11:**
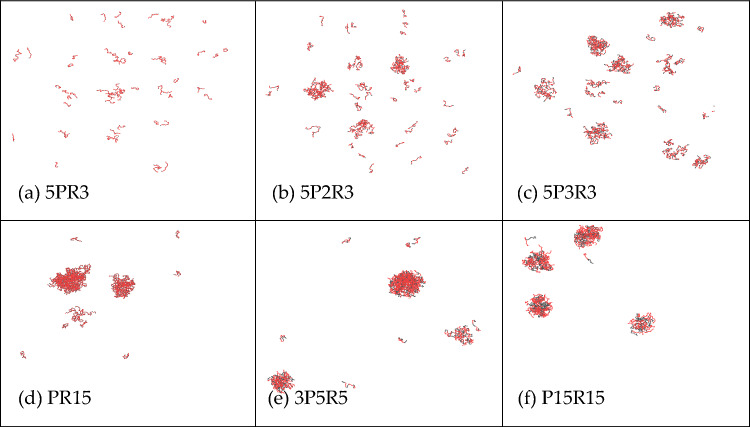
LLPS behavior of poly PR (1500 mM salt concentration, the total amount of arginine is 15) under different amino acid sequences.

Experiments have demonstrated that the alternating distribution of arginine is responsible for the toxicity of poly-PR^24^. In our investigation, we explored the impact of different arrangements of proline and arginine in PR15 on phase separation. Using the concentration at which PR15 initially exhibited phase separation, 1500 mM, as a prototype, we modified the arrangement of proline and arginine. Keeping the total number of arginine residues at 15, we simulated the outcomes of varying the number and arrangement of prolines in Fig. 11.

Figure 11 shows the LLPS behavior of poly PR with a total of 15 amino acids under different amino acid sequences. Experimental findings revealed that different sequences of proline–arginine exhibited distinct degrees of aggregation. With a fixed quantity of arginine, increasing the separation of prolines resulted in a more pronounced aggregation effect. Similarly, for the same total number of proline-arginine repeats, the outcomes varied significantly based on the location of arginine.

**Figure 12:**
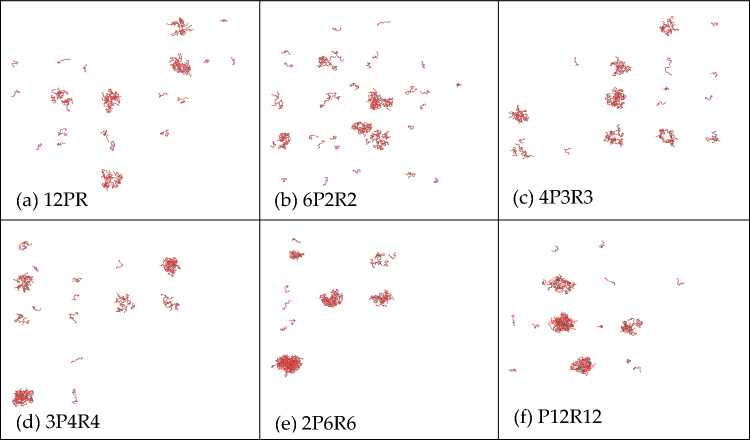
LLPS behavior of poly PR (1200 mM salt concentration, the total number of both amino acids is 12) under different amino acid sequences.

To delve deeper into the influence of amino acid sequence on phase separation, we simulated the phase separation results of different amino acid sequences of PR12 at a concentration of 1200 mM. Figure 12 shows the LLPS behavior of poly PR with a total of 12 amino acids under different amino acid sequences. The simulation results showed that a smaller number of proline-arginine repeats led to a more evident aggregation effect.

### Simulation and experimental verification of phase separation of GR15 and salmon sperm DNA

Additionally, to validate the reliability of the simulation program, we simulated the phase separation behavior of GR15 with single-stranded DNA, which bears an extremely similar composition to PR15. Subsequently, we conducted experimental verification. All reagents and chemicals were procured in the highest purity. Tris base was purchased from Sigma-Aldrich, and the Gly-Arg repeat sequence (GR15) was obtained in freeze-dried powder form from Jiangsu Jitaipeptide Industry Technology Co., Ltd. (Suzhou, China, Lot. No.: P231120-MX432173). Oligonucleotides (i.e., salmon sperm DNA) were obtained in dry form from Thermo Fisher Science US Inc., and purified water was acquired from the Milli-Q system.

The phase separation of GR15 with salmon sperm DNA was observed using a Nikon Ti-E inverted microscope equipped with a Nikon 50× oil-immersion objective (Nikon CFI Apo 50XW NIR) and a 48MP FHD Camera V8. As salmon sperm DNA carries a negative charge, glycine in GR15 is uncharged, and arginine carries a positive charge, we assumed that the mixed solution of GR15 and salmon sperm neutralized the electrostatic charges, canceling out the arginine charge.

**Figure 13:**
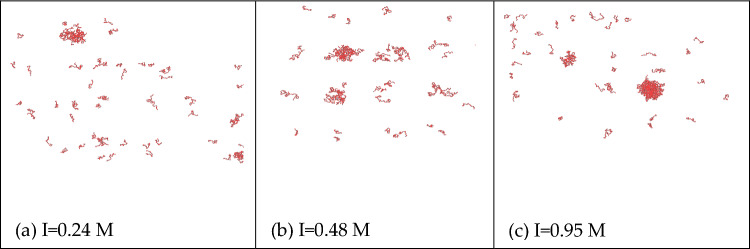
LLPS behavior of GR15 and salmon sperm DNA solutions with different ionic strengths.

**Figure 14:**
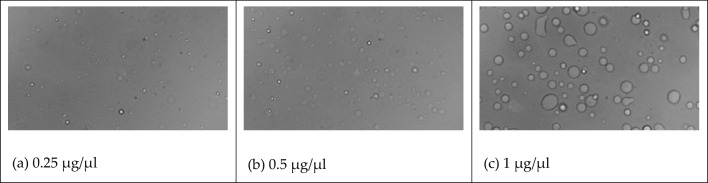
LLPS behavior of GR15 and salmon sperm DNA solutions with different mass concentrations.

As shown in Fig. 13, we simulated mixed solutions of salmon sperm with GR15 at concentrations of 0.25 μg/μl, 0.5 μg/μl, and 1 μg/μl, corresponding to calculated ionic strengths of 0.24 M, 0.48 M, and 0.95 M, respectively. The results demonstrated phase separation in all cases, with more prominent phase separation observed as the salmon sperm concentration increased. Figure 14 illustrates the experimental verification, wherein the droplets of phase-separated condensate became larger with an increase in salmon sperm DNA concentration. The experimental results are in substantial agreement with the simulation results, affirming the feasibility of our phase separation simulation program.

## Conclusions

In our comprehensive exploration, we delved into the LLPS behavior of poly-proline-arginine under different salt concentrations and temperatures, elucidating the roles of hydrophobic and electrostatic interactions in LLPS. Our key findings are summarized below:

- *Threshold for LLPS* LLPS behavior in PR peptides is observable only when the concentration surpasses 1000 mM, with the required salt concentration for LLPS escalating with sequence length. PR peptides with a repeat sequence of fewer than 10 did not manifest LLPS at any salt concentration. The occurrence of phase separation of PR peptides is only related to salt concentration and their length, and changes in the quantity of PR peptides do not affect the results of phase separation.
- *Influence of Interactions* Both hydrophobic and electrostatic interactions contribute to the LLPS behavior of PR peptides. Hydrophobic interaction exerts a significant impact, while electrostatic interaction plays a comparatively smaller role. In PR peptides exhibiting LLPS, reducing their hydrophobic value weakens or even eliminates LLPS, promoting homogeneity.
- *Temperature Impact* Temperature also influences the LLPS behavior of PR peptides. An increase in temperature leads to a decrease in the aggregation of PR15, and at 340 K, aggregation disappears, disrupting the LLPS behavior of PR15.
- *Sequence Variation* Different proline-arginine sequences in poly PR result in varying degrees of phase separation, emphasizing the significance of amino acid sequence in LLPS.
- Program Validation: Simulation and experimental verification of GR15 with salmon sperm DNA confirmed the program’s capability to predict phase separation behavior.

In conclusion, our simulation outcomes offer novel insights and methodologies for further understanding the potential mechanisms of LLPS. These findings may serve as a foundation for exploring innovative treatment strategies and drug development for diseases associated with phase separation phenomena.

## Data Availability

The data that support the findings of this study are available from the corresponding author, G. Y., upon reasonable request.
